# Insights into heme degradation and hydrogen peroxide-induced dimerization of human neuroglobin

**DOI:** 10.1042/BSR20241265

**Published:** 2025-01-21

**Authors:** Alice Cassiani, Paul G. Furtmüller, Marco Borsari, Gianantonio Battistuzzi, Stefan Hofbauer

**Affiliations:** 1 Department of Chemistry, Institute of Biochemistry, BOKU University, Muthgasse 18, A-1190, Vienna, Austria; 2 Department of Chemical and Geological Sciences, University of Modena and Reggio Emilia, via Campi 103, 41125 Modena, Italy

**Keywords:** covalent link, heme bleaching, human neuroglobin, oligomerization, protein aggregation

## Abstract

In this present study, we investigated the H_2_O_2_-induced oligomerization of wild-type human neuroglobin (hNgb) and of some selected variants (C46AC55A, Y44A, Y44F, Y44AC46AC55A, Y44AC46AC55A) to clarify how the process is affected by the Cys46/Cys55 disulfide bond and the distal H-bonding network and to figure out the molecular determinants of the H_2_O_2_-induced formation of amyloid-type structures and hNgb aggregates. It turns out that hydrogen peroxide exerts a two-fold effect on hNgb, inducing both heme breakdown and protein dimerization/polymerization. The enhanced resistance to the oxidizing effect of H_2_O_2_ of the disulfide-free variants indicates that both effects are strictly influenced by the heme accessibility for H_2_O_2_. Most importantly, the H_2_O_2_-induced neuroglobin dimerization/polymerization turns out to be triggered by tyrosyl radicals resulting from the oxidizing action of Compound I ([Por^•^Fe(IV) = O]^+^). Peptide mapping indicates that the H_2_O_2_-induced dimerization/polymerization of hNgb mainly involves Tyr44, which forms covalent bonds with all the other tyrosine residues, with a minor contribution from Tyr88. The presented findings contribute further important pieces of information in the quest of identifying all capabilities of hNgb and ultimately its physiological task.

## Introduction

Neuroglobin (Ngb) is a heme protein belonging to the class of globins. Its presence in mice and human neuronal tissues was first recognized in 2000 [1] and was later confirmed in different organisms, including mammals, fishes, avians, and amphibians [2]. Human neuroglobin (hNgb) is a monomeric protein of 151 amino acids with a molecular weight of 16933.41 Da. The protein consists of eight α-helices embedding the heme prosthetic group. The A, B, E, F, G, and H α-helices are organized into a two-layer structure [3,4], forming a ‘three-over-three α-helical sandwich’ structure, typical of the ‘globin fold’ [5,6]. However, Ngb displays a bis-histidyl six-coordinated heme *b*, since the heme-Fe is six-coordinated by four pyrrolic N-atoms of the tetrapyrrolic ring and the imidazole nitrogen atoms of the side chains of the distal (His64) and proximal (His96) histidine residues. hNgb is abundant in the neurons of the hypothalamus and in the retinal cells, and it is found in smaller amounts in other parts of the nervous central system [1,7–10].

The physiological role of Ngb and the mechanisms of its reactivity are still under debate [11,12]. All proposed functions that involve the binding of a ligand require the distal histidine dissociation from the hexacoordinated heme-Fe. The metal center in six coordinated globins is not directly accessible and rules out the possibility of the simple bimolecular reaction occurring in five coordinated hemoproteins. As a consequence, the rate of His64 coordination bond dissociation is limiting for the binding with exogenous ligands in Ngb [2,5,13,14]. Although an oxygen transport role for Ngb was initially suggested [4,5,9,15], the high autooxidation rate of ferrous Ngb and the slow dissociation rate of the distal histidine rule out the involvement of the protein in storage and transport of oxygen molecules [7,8,16].

More recent studies suggest that Ngb exerts a neuroprotective function [11,12]. Even if this physiological role is still a matter of discussion, evidence has been acquired that Ngb is a stress-inducible protein that is over-expressed under oxidative stress and in case of hypoxia and glucose deficiency [2,5,17–21]. It has been suggested that hNgb would have a positive effect against neurodegenerative disorders, such as Alzheimer’s disease [22–25] and glaucoma [26]. A regulatory role of the ‘sleep-wake-cycle’ of mammals is also under discussion [27]. The neuroprotective function could be related to the known ability of Ngb to act as a scavenger for harmful reactive oxygen (ROS) and nitrogen (NOS) species [28–31]. Other possible functions include the conversion of NO (nitric oxide) surplus to nitrate [32] and the production of NO from nitrite anion for signaling events [30,33–35]. Ngb has also been reported to interact with various proteins. Its interaction with cytochrome *c* is of particular interest. The ferric form of cytochrome *c* (cyt *c*) is one of the initiators of cellular apoptosis when released from the mitochondria into the cytosol, in response to a stress challenge. It has been shown that Ngb can reduce the ferric form of cyt *c*, thus preventing the apoptotic cascade [2,36–40].

**Figure 1:**
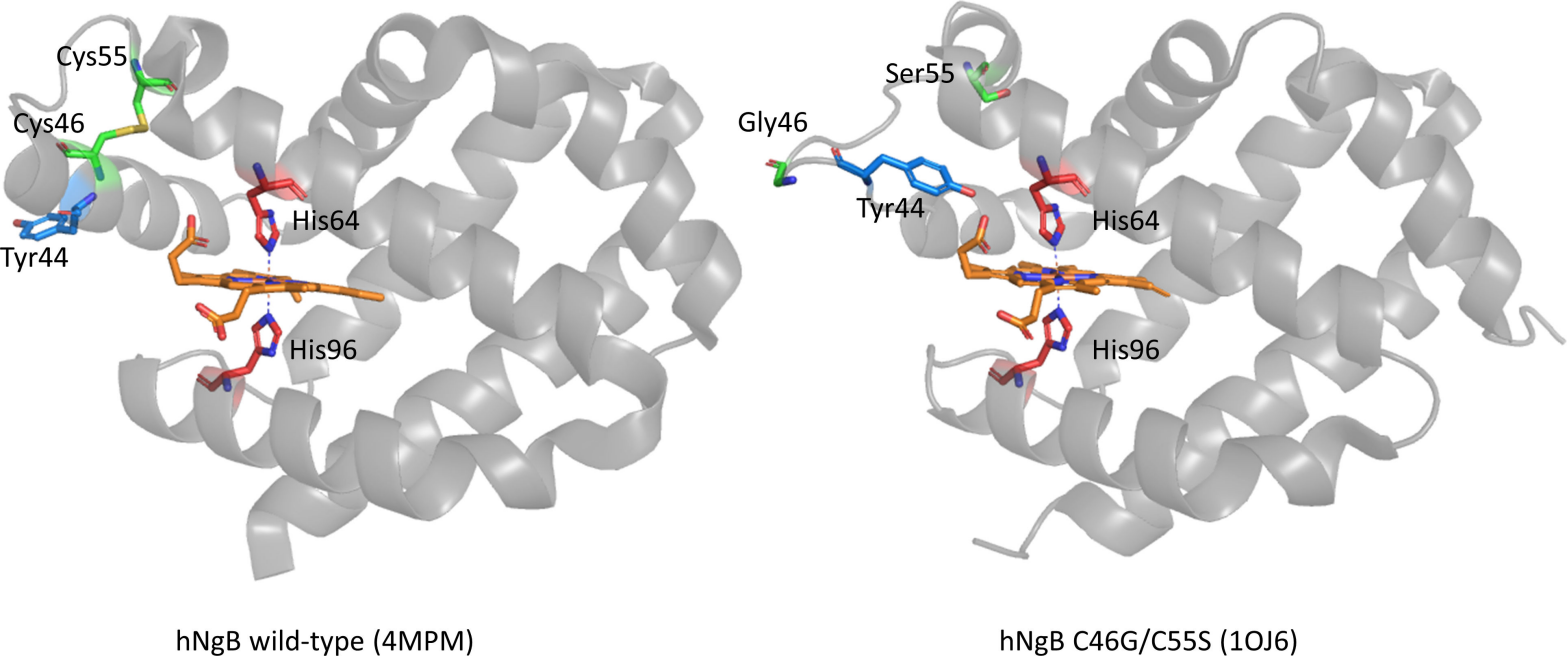
Three dimensional structure of wild-type human neuroglobin (hNgb) and its C46GC55S mutant.

hNgb features an intramolecular disulfide bridge connecting cysteines Cys46 and Cys55, whose formation is regulated by the surrounding cellular environment: under cytosolic (reducing) conditions, the disulfide bond is not present as both Cys residues are in their reduced form, whereas under oxidative stress conditions, the S-S bond is formed. Cleavage of the disulfide bond alters the conformation of the CD loop (residues 36–59), connecting the helices C and D, which shifts from an α−helical structure to a β-turn followed by a short distorted β-strand formed by residues 42–49 (Figure 1) [3,4]. This structural rearrangement deeply modifies the H-bonding network involving the heme propionates, without significantly altering the heme cavity and the three-dimensional structure of the remaining portions of the protein [3,4]. The structural effect of the cleavage of the disulfide bridge (due to oxidation or mutation of Cys46 and Cys55) strengthens the bond between the heme iron and the distal histidine, lowering the affinity of hNgb for exogenous ligands [2,3,15,31,41–45]. Conversely, experimental and computational studies confirmed that the presence of the disulfide bridge increases the dissociation rate of the distal histidine, thereby increasing both the affinity for exogenous ligand and the activity as NO_2_^−^ reductase [34]. Hence, it appears that the *in vivo* functionality of hNgb is modulated by the Cys46/Cys55 disulfide bridge [3,4], which connects the ability of the protein to bind exogeneous ligands to the redox state of the cell [3,4].

Cartoon representation of the structure of wild-type human neuroglobin (hNgb) (4mpm.pdb, chain A) featuring the disulfide bridge between Cys46 and Cys55 (left) and of the C46G/C55S mutant of hNgb (1oj6.pdb, chain B) without the disulfide bridge between Cys46 and Cys55 (right). The heme group is shown in orange, and the iron axial ligands His 64 and His 96 are represented in red, whereas the residues that were mutated in the present study are represented in green (Cys 46 and Cys 55) and blue (Tyr 44).).

In the present study, we investigated the H_2_O_2_-induced oligomerization of wild-type hNgb and of some selected variants, featuring different accessibility of the heme center, to clarify the molecular details influencing the H_2_O_2_-induced formation of amyloid-type structures and hNgb aggregates previously reported [2,5,17–21]. Understanding the effect exerted on the structure of hNgb by an oxidizing agent that is formed under oxidative stress conditions would increase our knowledge of the molecular determinants influencing its protective role against oxidative cell damage.

Two different groups of mutations were considered: one mutation targets the replacement of cysteines Cys46 and Cys55 with two alanines (C46AC55A), resulting in the deletion of the Cys46/Cys55 disulfide bridge [2,5,17–21], and the other involves the replacement of tyrosine 44 with an alanine (Y44A) or a phenylalanine (Y44F) (Figure 1) [2,5,17–21]. Hence, a total of five hNgb variants were studied (C46AC55A, Y44A, Y44F, Y44AC46AC55A, and Y44FC46AC55A).

As Tyr44 belongs to the CD loop, its spatial orientation changes depending on the presence or the absence of the disulfide bridge. In the presence of the S-S bond, Tyr44 moves away from the distal zone, whereas in the absence of the S-S bridge, as in the case of the C46AC55A mutant, Tyr44 moves closer to the heme, pointing toward the heme distal cavity [46]. In the latter case, Tyr44 is involved in an H-bonding and electrostatic network, which includes one of the heme propionates (propionate 7) and the distal His64 [3,47] and exerts a strong stabilizing effect on the heme environment, limiting the access of exogenous ligand to the heme-Fe (Figure 1).

The Y44FC46AC55A and Y44AC46AC55A triple mutations should result in a significant alteration of this network of interaction, due to the different size of alanine and phenylalanine side chains and their inability to form H-bonds. Indeed, the former triple mutation modifies the distal H-bonding network without significantly affecting the heme accessibility, whereas the Y44AC46AC55A variant couples an altered H-bonding network with an increased heme accessibility, due to the limited steric hindrance of the Ala methyl group, compared with the aromatic groups of Tyr and Phe. For the same reasons, the single Y44A and Y44F mutations affect the α-helical structure assumed by the CD loop in the presence of the Cys46-Cys55 S-S bond.

It turns out that hydrogen peroxide exerts a two-fold effect on hNgb, inducing heme breakdown and protein dimerization/polymerization. The enhanced resistance to the oxidizing effect of H_2_O_2_ of the disulfide-free variants indicates that both effects are strictly influenced by the heme accessibility by H_2_O_2_. Most importantly, the H_2_O_2_-induced Ngb dimerization/polymerization turns out to be triggered by tyrosyl radicals resulting from the oxidizing action of Compound I ([Por^•^Fe(IV) = O]^+^) [48]. Peptide mapping unambiguously shows that the hNgb dimerization/polimerization mainly involves Tyr44, which forms covalent bonds with all the tyrosine residues, with a minor contribution from Tyr88; this finding adds significantly to the understanding of hydrogen peroxide mediated cross-linking in hNgb. With this investigation, new bits and pieces about hNgb are described and studied, which will help identify its physiological task and relevance.

## Methods

### Expression and purification

Wild-type hNgb and the five Y44A, Y44F, C46AC55A, Y44AC46AC55A, and Y44FC46AC55A mutants were expressed in *Escherichia coli* and purified as previously reported in Di Rocco et al. [21].

### Spectroscopic studies

Electronic absorption spectra were recorded with a HITACHI U3900 UV-vis spectrophotometer. All experiments were carried out with 5 μM protein solutions in 50 mM phosphate buffer pH 7. The protein concentration was calculated with Lambert-Beer law, using extinction coefficient of ε_412_ = 129,000 M^**−**1^ cm^**−**1^. Titration with cyanide was performed on the above solutions until no change in the absorption spectrum in correspondence with the Soret (around 412 nm) and Q (532 nm and 554 nm) bands was observed. The plot of the absorbance at λ_max_ against cyanide concentration (µM) was fitted to obtain the *K*_D_ values.

### HPLC-SEC-PDA-MALS (High Performance Size Exclusion Liquid Chromatography-Photo Diode Array-Multi Angle Light Scattering) measurements

All analyses were performed using an LC20 prominence HPLC system equipped with the refractive index detector RID-10A, the photodiode-array detector SPD-M20A, and a MALS Heleos Dawn8+plus QELS 12 detector. The column (Superdex 200 10/300 GL, GE Healthcare, Chicago, Illinois, U.S.A.) was equilibrated with 1 × PBS (pH 7.4) as running buffer, at a flow rate of 0.75 mL·min^-1^ at 25°C. Chromatograms were recorded at 412 nm (Soret band), and the molecular weight of the eluted peaks was determined by MALS detector using ASTRA 6 software (Wyatt Technology, Santa Clara, California, U.S.A.).

Then, 1 μg/μL protein solutions incubated for 20 h at 4°C with a different excess of H₂O₂ (calculated as molar ratio H_2_O_2_/protein) were centrifuged at 13,000 rpm for 2 min at room temperature and filtered with 0.1 mm Ultrafree-MC-VV filter (Merck Millipore, Darmstad, Germany), and a total amount of 70 μL was injected for each chromatographic run.

For spin trapping experiments on the wild-type hNgb, 5 mg MNP were dissolved in 100 µL H₂O₂ and heated up to 60°C in the dark. The assay with 1 µg/µL protein and 10 µg/µL MNP was incubated with a 15-fold of H_2_O_2_ for 20 h at 4°C.

### Peptide mapping

Dimeric wild-type hNgb and Y44A variant were isolated after incubation with 15-fold and 17-fold excess of H₂O₂, respectively. The purification was performed by size-exclusion chromatography (HiLoad 16/600, Superdex 75). All solutions were concentrated in Amicon Ultra Centrifugal Filter Units. LC-ESI-MS/MS (Liquid Chromatography-Electro Spray Ionization-Tandem Mass Spectrometry) analysis of peptides originating from protease cleavage was performed as follows. The proteins were S-alkylated with iodoacetamide and digested with Trypsin (Promega). The digested samples were loaded on a nanoEase C18 column (nanoEase M/Z HSS T3 Column, 100 Å, 1.8 µm, 300 µm **×** 150 mm, Waters) using 0.1% formic acid as the aqueous solvent. A gradient from 1% B (B: 80% acetonitrile, 0.1% FA) to 40% B in 50 min was applied, followed by a 10-min gradient from 40% B to 95% B that facilitates elution of large peptides at a flow rate of 6 µL/min. Detection was performed with an Orbitap MS (Exploris 480, Thermo) equipped with the standard H-ESI source in positive ion, DDA (Data Dependent Acquisition) mode ( = switching to MS/MS mode for eluting peaks). MS scans were recorded (range: 350–1200 Da), and the 20 highest peaks were selected for fragmentation. Instrument calibration was performed using Pierce FlexMix Calibration Solution (Thermo Scientific). The analysis files were analyzed using PEAKS, which is suitable for performing MS/MS ion searches. The files were searched against a *E. coli* database, containing the sequence of the target protein. Additionally, the files were analyzed manually to identify potential Tyrosine linkages by FreeStyle 1.8 (Thermo Scientific).

## Results

**Figure 2:**
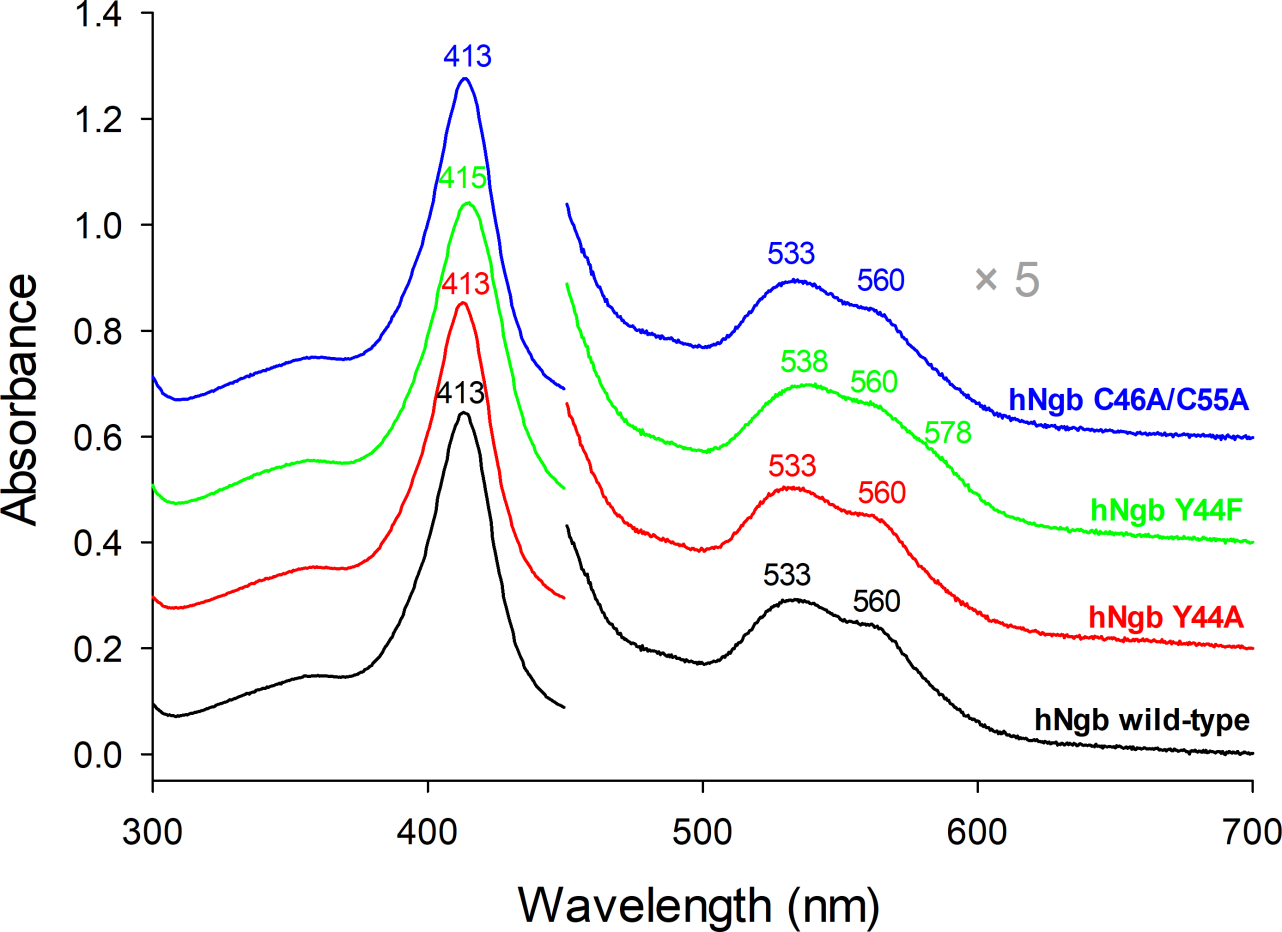
UV-vis spectra of wild-type human nuroglobin (hNgb) and its Y44A, Y44F and C46AC55A variants.

Although the physiological role of hNgb is still not fully understood, there are indications that the accessibility of heme *b* and its protein environment is crucial in defining its functional properties [21,44]. Therefore wild-type hNgb and selected variants were investigated at a molecular level, to get a solid basic understanding of structure-function relationships. Even though the mutations of the variants are close to the active site and the heme *b* cofactor, the UV-vis absorption spectra of the variants Y44A, Y44F, and C46AC55A and their combinations hardly differ from the wild-type (Figure 2) [21]. Wild-type, Y44A, C46AC55A, and Y44AC46AC55A exhibit UV-vis absorption spectra characteristic of a bis-histidine ligated low-spin heme with the Soret band at 413 nm and Q-bands at 533 nm and 560 nm and no charge transfer band. This is also the dominating spectral species in the Y44F and the Y44FC46AC55A variants, albeit in these samples, a second low-spin species is present, causing a 2–3 nm red shift of the Soret band (415–416 nm) and the appearance of a shoulder at 578–580 nm, indicative of an OH^−^ ligation [49].

UV-vis absorption spectra of human neuroglobin (hNgb) wild-type (black), and variants Y44A (red), Y44F (green), and and C46AC55A (blue) , Y44AC46AC55A (magenta), Y44FC46AC55A (cyan) (50 mM phosphate buffer, pH 7.0). Spectra are presented with a base- shift of 0.2 along the y-axis, and the visible region (450–700 nm) is enlarged five-fold for better visibility.y.

**Figure 3:**
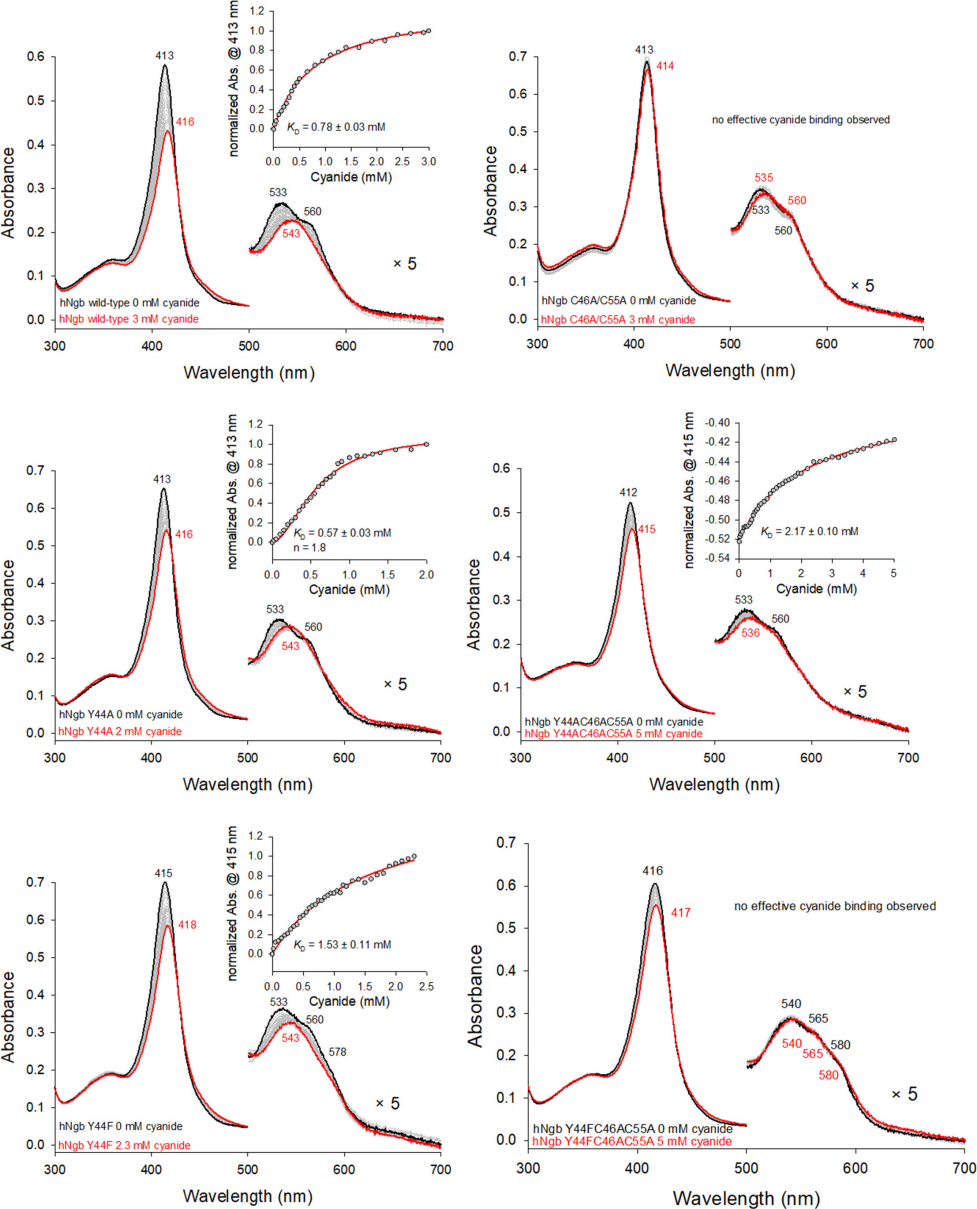
Cyanide binding to human neuroglobin (hNgb) wild-type and variants.

In order to rationalize the observed differences of hNgb wild-type and variants with the substrate hydrogen peroxide [21], the active site accessibility for exogenous molecules and the strength of the bond connecting the Fe(III)-heme and the distal His64 were tested by ligand binding studies using cyanide. The obtained *K*_D_ values clearly indicate the importance of the disulfide bond for the integrity of the active site and the heme accessibility. The wild-type protein and the Y44F variant exhibit a *K*_D_ value for cyanide of 0.78 mM and 1.53 mM, following a hyperbolic model. Interestingly, cyanide binding to Y44A could be fitted best using a Hill equation, yielding a *K*_D_ value of 0.57 mM and a Hill coefficient of 1.8, which indicates cooperativity. Although the observed cooperativity is difficult to explain in a monomeric system, it could arise from an equilibrium between two forms of hNgb Y44A, featuring an open and a closed conformation, respectively, leading to different active site accessibilities within one sample. The initial binding of cyanide could shift the equilibrium, resulting in the positive cooperativity observed. Possibly, dimerization might lead to the observed cooperativity of cyanide binding in this variant. No cyanide binding could be detected for the disulfide bond-free C46AC55A variant and the triple variant Y44FC46AC55A, whereas the Y44AC46AC55A mutant exhibits a *K*_D_ value for cyanide of approximately 2.2 mM. The inability of the C46AC55A and Y44FC46AC55A variants fits with the strengthening of the Fe(III)-His64 bond and the reduced accessibility of the heme distal site, resulting from the deletion of the C46-C55 disulfide bridge [2,3,15,31,41–45]. Hence, cyanide binding appears not to be significantly influenced by the alteration of the H-bonding network connecting the OH group of Tyr44, heme propionate 7, and the distal His64 in the Y44FC46AC55A variant [3,47]. The higher affinity of the Y44AC46AC55A mutant for the cyanide ion can be explained by the limited steric hindrance of the alanine sidechain, which is too small to completely shut off cyanide binding, despite the spatial reorganization due to the loss of the disulfide bond (Figures 1 and 3).

UV-vis spectra at zero cyanide are depicted as black lines, and final spectra at highest cyanide concentrations as red lines. Intermediate spectra are shown as grey lines. The insets depict the plots of absorbance change at the Soret maximum *versus* the cyanide concentration with corresponding hyperbola fits (for wild-typewild-type, Y44F, and Y44AC46AC55A) and a sigmoidal fit for Y44A to derive kinetic parameters.s.

**Figure 4:**
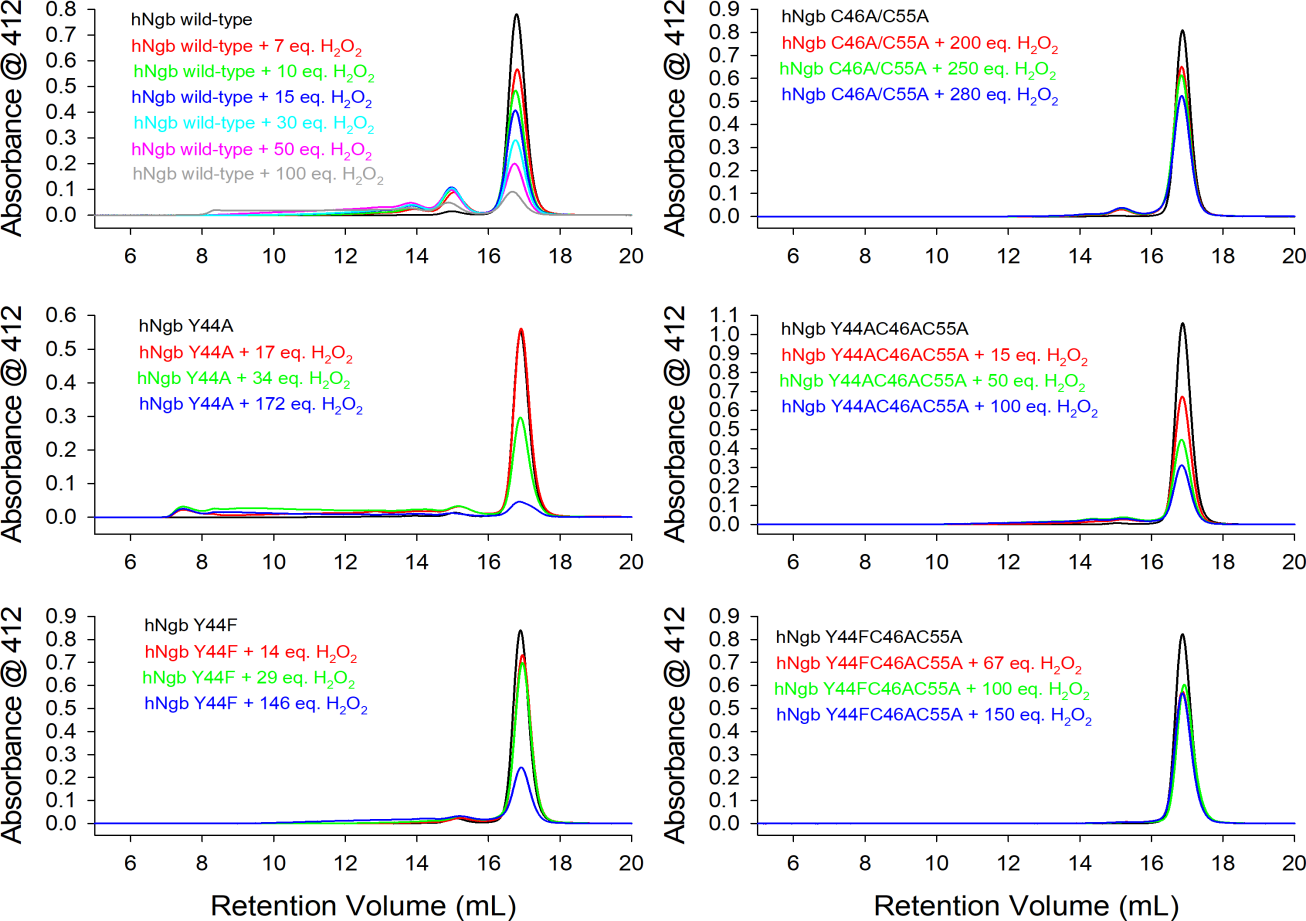
HPLC-SEC (High Performance Size Exclusion Liquid Chromatography) profiles of human neuroglobin (hNgb) wild-type and variants, treated with hydrogen peroxide.

**Figure 5:**
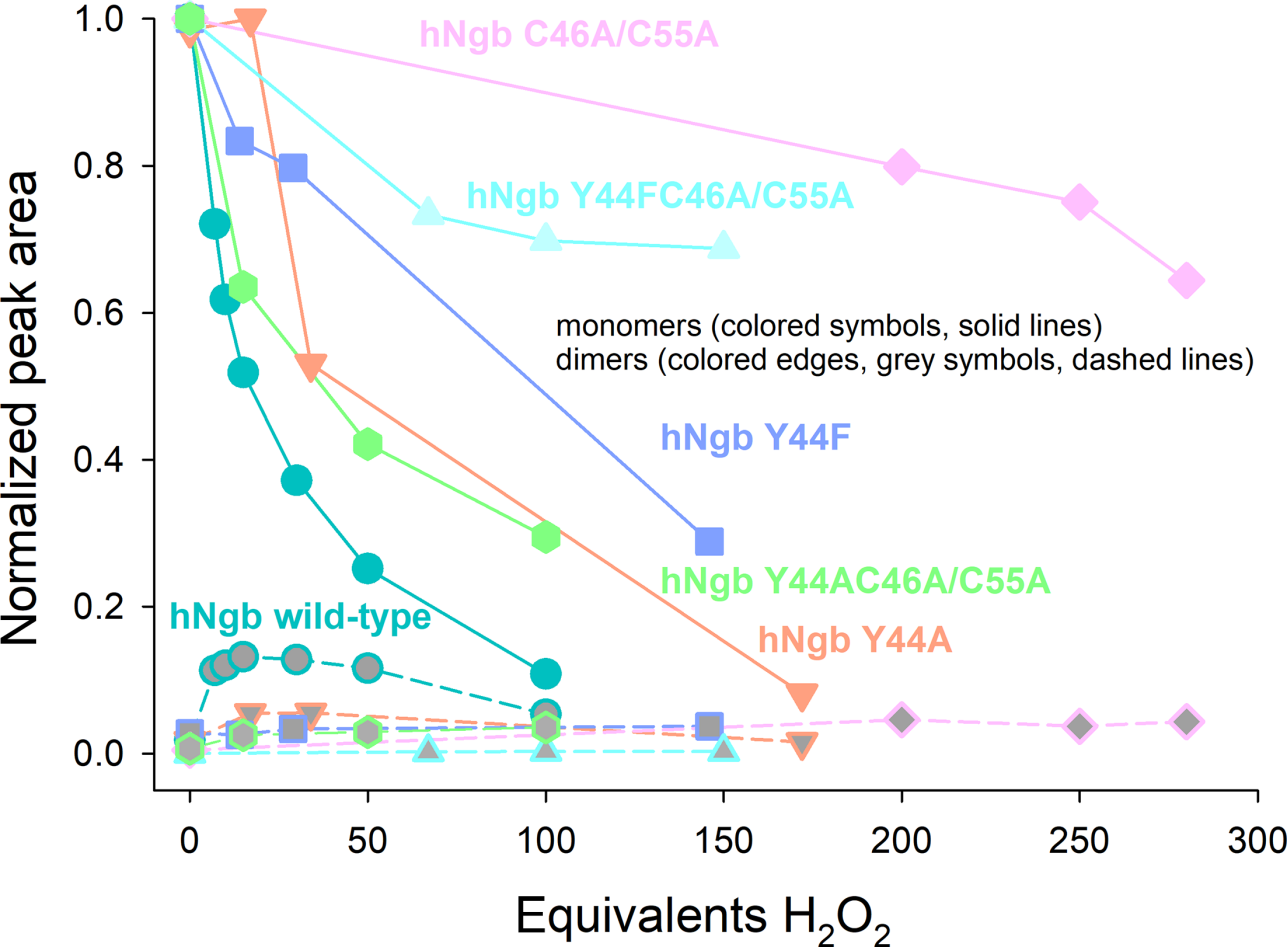
Relative quantification of monomeric and dimeric wild-type human neuroglobin and its Y44A, Y44F, C46AC55A, Y44FC46AC55C, Y44AC46AC55A variants in the presence of increasing H_2_O_2_ concentrations.

The oligomerization of wild-type hNgb and its variants upon treatment with hydrogen peroxide was analyzed by HPLC-SEC-MALS (High Performance Size Exclusion Liquid Chromatography with Multi-Angle Light Scattering). The HPLC-SEC-MALS profiles reported in Figure 4 show that two phenomena invariably occur, although their relative amount is protein specific: (i) hNgb dimerization/polymerization (indicated by the peaks with a retention volume ≤ 15 mL) and/or (ii) heme bleaching of the monomeric form (indicated by the decrease of peak with a retention volume of 17 mL) as well as of the dimers. The normalized peak areas for wild type Ngb and its mutants in the presence of increasing concentration of H_2_O_2_ are reported in Figure 5.

The absorbance at 412 nm (Soret region) is plotted *versus* Thethe retention volume. The respective excess of hydrogen peroxide as well as the color- code of the samples are reported in the figure..

Relative quantification of monomeric (full symbols) and dimeric (grey symbols with colored edge) human neuroglobin (hNgb) wild typewild-type (turquoise) and variants (Y44A, orange; Y44F, blue; C464AC55A, pink; Y44FC46AC55A, cyan; Y44AC46AC55A, green) as a function of hydrogen peroxide excess analyzed by HPLC-SEC (High Performance Size Exclusion Liquid Chromatography).

The normalized area of peak corresponding to monomeric wild-type hNgb greatly decreases up to a 100-fold excess of H₂O₂, indicating an extensive heme bleaching, while the normalized area of the peak of its dimeric form increases up to a 15-fold excess of H₂O₂ and decrease in the presence of higher concentrations of H₂O₂ (Figure 5). Other aggregates with higher molecular mass are also observed, although their low amount prevented the determination of their molecular mass.

No dimerization and only a limited heme bleaching at a very high excess of H₂O₂ are observed for the C46AC55A and the Y44FC46AC55A variants, whereas the Y44AC46AC55A mutant is more prone both to dimerization and heme bleaching (Figure 5). Hence, it appears that the reduction of heme accessibility resulting from the structural reorganization induced by the deletion of the disulfide bridge greatly enhances the endurance of Ngb to the oxidizing action of hydrogen peroxide [2,21]. The increased reactivity of the Y44AC46AC55A mutant compared with the C46AC55A and Y44FC46AC55A variants confirms the importance of heme accessibility, as the alanine sidechain is too small to effectively prevent the access of H_2_O_2_ to the heme cavity.

Although no information is currently available concerning the redox behavior of the Compound I/Compound II redox couple in native or mutated Ngb, its *E*°’ value is influenced by the same molecular factors determining that of the Fe(III)/Fe(II) redox couple [50,51]. Therefore the *E*°’ values of the Fe(III)/Fe(II) couple in wild-type hNgb and its alkylated derivative (hNgbSAlk), which mimics the C46AC55A mutant, provide some indication about the redox reactivity of the catalytically relevant redox couple [44]. Cleavage of the disulfide bond exerts a negligible effect on the *E*°’ values of the Fe(III)/Fe(II) couple (Δ*E*°’ = −5 mV) [44]; hence, it is likely that it does not significantly impact on that of the Compound I/Compound II redox couple too, resulting in similar *E*°’ in wild-type hNgb and the C46AC55A mutant. Since the former dimerizes, whereas the latter does not, it appears that no direct relationship exists between the *E*°’ of the Compound I/Compound II redox couple and the amount of the observed H_2_O_2_-induced dimerization.

Both Y44A and Y44F mutants show some dimerization and a very limited heme bleaching up to a 17-fold excess of hydrogen peroxide, whereas extensive heme bleaching occurs in the presence of a very large excess of H₂O₂. Hence, the replacement of Tyr44 with an alanine or a phenylalanine greatly reduces H_2_O_2_-induced dimerization and heme bleaching compared with wild-type hNgb (Figures 4 and 5). This observation indicates that, in addition to the accessibility of the active site, a dimerization mechanism occurs in which Y44 is important. Tyrosyl radicals can be produced upon reaction of hNgb with hydrogen peroxide to form and oxidize porphyryl radical (Compound I) and further a tyrosyl radical (Compound I*). These tyrosyl radicals can build covalent linkages and lead to protein dimerization [52–56].

**Figure 6:**
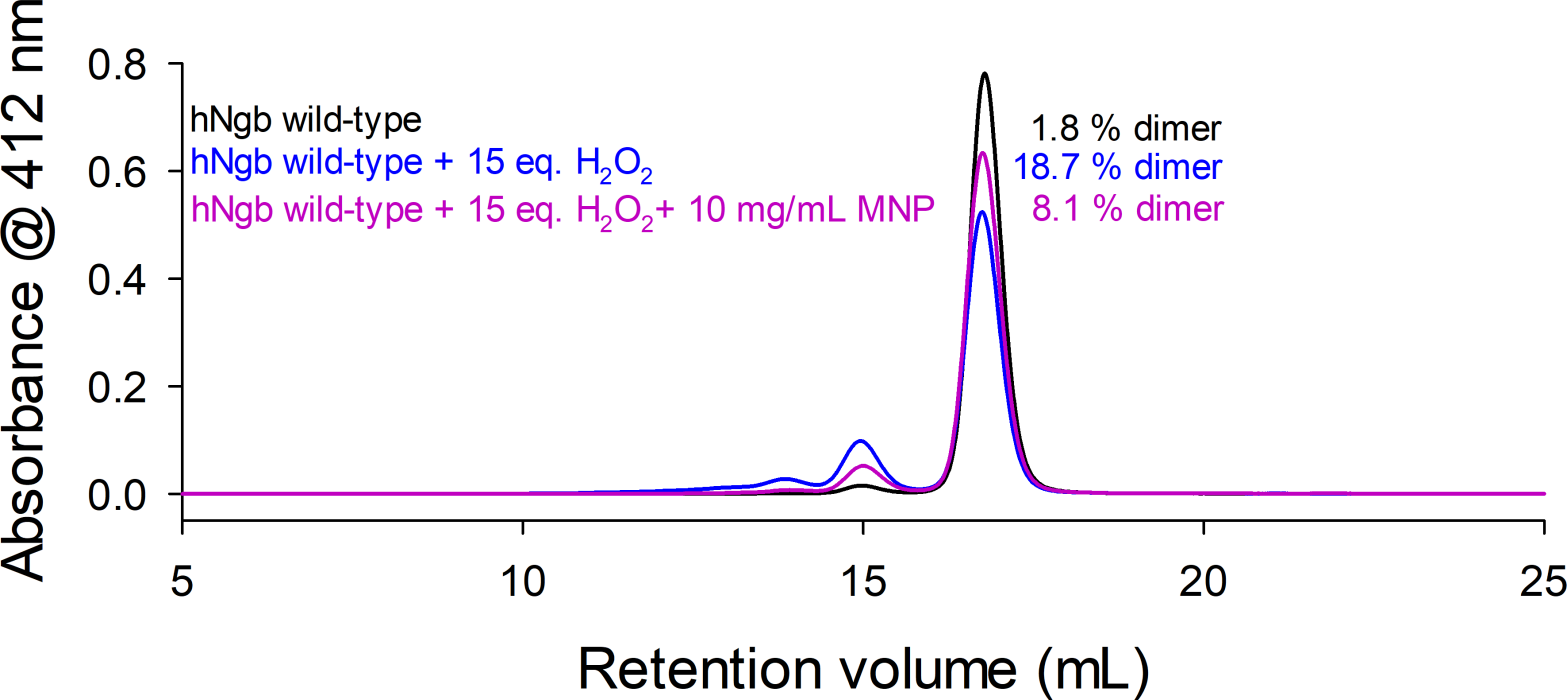
HPLC-SEC profiles of wild-type human neuroglobin and in the presence of H_2_O_2_ and H_2_O_2_+MNP

2-Methyl-2-nitrosopropane (MNP) is a spin trap that specifically attacks and modifies tyrosyl radicals yielding 3-nitrotyrosine [57]. Although the reaction kinetics of this process are difficult to follow or estimate, a qualitative assessment is possible and presented in Figure 6. It turns out that when MNP is present in the reaction mixture of wild-type hNgb and a 15-fold excess of hydrogen peroxide, a significant reduction of dimerization (8.1% in the presence of MNP, 18.7% in the absence of MNP) is observed, clearly pointing out the involvement of tyrosyl radicals in the dimerization process.

HPLC-SEC (High Performance Size Exclusion Liquid Chromatography) profiles of wild-typewild-type human neuroglobin (hNgb) (black) and in the presence of hydrogen peroxide (blue) and MNP2-methyl-2-nitrosopropane (MNP) and hydrogen peroxide (magenta).).

**Figure 7:**
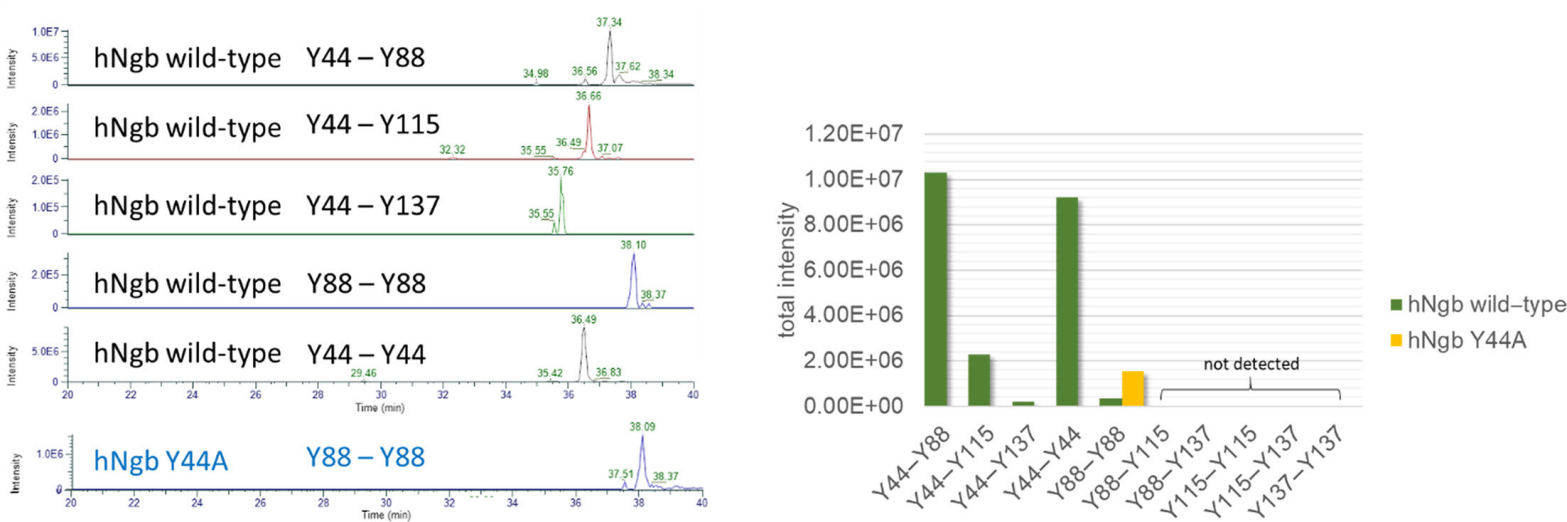
Mass spectrometry data of dimeric wild-type human neuroglobin and of its Y44A variant.

Wild-type hNgb has four tyrosine residues (Y44, Y88, Y115, and Y137). To identify the tyrosine residues responsible for dimerization, dimeric fractions of the hydrogen peroxide treated wild-type hNgb samples were purified by preparative size-exclusion chromatography and were analyzed by mass spectrometry (peptide mapping). Figure 7 summarizes the obtained results, which clearly show that Y44 is contributing the most to dimerization by forming linkages to all other tyrosine residues of the other monomer (confirmed by MS2 spectra, Supplementary Figure S1), especially to Y88 and Y44. Additionally, also Y88 can form a covalent linkage with Y88 of another monomer. This Y88–Y88 linkage is also observed in the Y44A variant and explains the small amount of detected dimerization (Figures 4 and 5) in this variant.

Mass spectrometry data of purified, dimeric human neuroglobin (hNgb) wild-typewild-type and Y44A variant (right). On the left, total intensities of identified linked peptides are presented.

## Discussion

The hNgb has been observed to form oligomers upon the presence of radical oxygen species, and this aggregation behavior might be linked to its physiological function, which is not still fully understood [2,21]. Tyr44, which is part of the CD loop, has been a target of investigation, being involved in the distal H-bonding network when the C46C55 disulfide bridge is not present [21,46]. In the present study, we studied the role of Tyr44 in the H_2_O_2_-induced oligomerization of hNgb, analyzing the behavior of selected protein variants, in which Y44 was replaced with a small alanine or bulky phenylalanine, in comparison with the wild-type protein. Beyond the Y44A and Y44F mutants, still conserving the disulfide bond connecting Cys46 to Cys55, three variants lacking the disulfide bond (C46AC55A, Y44AC46AC55A, and Y44FC46AC55A) were analyzed. Although the overall structural and spectroscopic features seem to be conserved (Figure 2), the access to the heme cavity is severely hampered in the disulfide-free variants, in which cyanide binding was significantly reduced or completely impaired, as in the case of C46AC55A and Y44FC46AC55A species. Cyanide binding to the Y44A and Y44F variants was still possible and only varied according to the size of the phenylalanine and alanine side chains (Figure 3). This is further reflected in the dimerization behavior of all samples upon reaction with hydrogen peroxide. Only the wild-type protein and the Y44A variant produced a significant amount of dimeric forms, while in the other variants, the dimerization process was almost completely inhibited (Figures 4 and 5). Analogously, the most extensive heme bleaching was observed in the wild-type and the Y44A variant (Figure 5). The limited reactivity of the disulfide-free variants can be explained by the rearrangement of the CD loop, which reduces the accessibility of the metal site for the incoming substrate (hydrogen peroxide) and strengthens the bond between the ferric heme and the distal histidine (His64) bridge [2,3,15,31,41–45]. Analogously, the reduced reactivity of the Y44F variant compared with the wild-type protein and the Y44A mutant can arise, at least in part, from the lower accessibility of its metal site (Figures 4 and 5).

These initial results raised questions about the mechanism of dimerization and the role of Tyr44. The formation of covalent linkages between subunits of heme proteins is often facilitated by oxidative mechanisms triggered by hydrogen peroxide [58,59]. Consequently, the two-electron deficient Compound I ([Por^•^Fe(IV) = O]^+^) is formed that can react oxidatively to form tyrosyl radicals leading to a Compound I* (Por^•^Fe(IV) = O … AA^•^) species. These tyrosyl radicals can ultimately form covalent linkages resulting in the observed dimerization of hNgb. Indeed, the dimerization of hNgb is greatly reduced by the spin trap MNP, which reacts with tyrosyl radicals, blocking the dimerization reaction (Figure 6), indicating that radical tyrosyl species are involved. Full inhibition of dimerization is not achieved, possibly due to different reaction kinetics of the reaction of tyrosyl radicals with MNP and the dimerization process. It has to be mentioned that the different kinetics cannot be determined reliably as MNP quantification and assay conditions are difficult to follow. Nevertheless, the qualitative message obtained by these results supports the involvement of tyrosyl radicals in the H_2_O_2_-induced dimerization of hNgb. This supports a previous study that has identified tyrosyl radicals of Tyr88 in the H64V variant of hNgb [48]. hNgb has four tyrosine residues (Tyr44, Tyr88, Tyr115, and Tyr137). Mass spectrometry and peptide mapping of dimeric hNgb samples show that Tyr44 is the main responsible for dimerization, as it forms covalent linkages to all four tyrosine residues of the other subunit (mainly to Tyr44 and Tyr 88) (Figure 7). The only identified linkage not involving Y44 was established between Tyr88 belonging to two different hNgb monomers. This bond was also the only detected linkage in the purified Y44A dimer, explaining why Y44A is still able to dimerize to some extent. Y44F and the disulfide-free variants do not show significant dimerization behavior, most probably because of the impaired accessibility for hydrogen peroxide, which minimizes heme *b* oxidation to Compound I.

## Conclusion

The present study shows that the oxidizing action of hydrogen peroxide exerts a two-fold effect on hNgb, resulting in heme breakdown and protein dimerization/polymerization. It turns out that both effects are strictly related to the heme accessibility by H_2_O_2_, as indicated by the enhanced resistance to H_2_O_2_ of the disulfide-free variants, whose metal center features a sensibly decreased solvent accessibility compared with wild-type hNgb. Most importantly, the presented data unequivocally show that H_2_O_2_-induced Ngb dimerization/polymerization is triggered by tyrosyl radicals resulting from the oxidizing action of Compound I ([Por^•^Fe(IV) = O]^+^). Tyr44 is the main responsible for the observed dimerization, forming covalent bonds with all the tyrosine residues of other protein molecules.

Therefore, it appears that under oxidative stress conditions, Ngb may exert two simultaneous and opposite functions: (i) it can protect the cell by consuming ROS through heme degradation, although the existence of possible redox partners able to reduce Compound I, thus avoiding heme degradation *in vivo*, cannot be ruled out, and (ii) it can undergo to radical polymerization, resulting in the formation of protein aggregates [19], possibly contributing to the onset of neurodegenerative diseases. Identification of the role of Tyr44 in the H_2_O_2_-induced dimerization provides an important piece of knowledge to better understand the molecular basis of the physiological role and the reactivity of hNgb in oxidative stress conditions.

## Supplementary material

Figure S1

## Data Availability

Data is available in the zenodo-repository of BOKU University (doi: 10.5281/zenodo.13707505).

## Abbreviations

MNP: 2-Methyl-2-nitrosopropane
Ngb: neuroglobin
cyt *c*: cytochrome *c*
hNgb: human neuroglobin

## References

[1] Burmester T., Weich B., Reinhardt S., Hankeln T (2000). A vertebrate globin expressed in the brain. Nat. New Biol..

[2] Ascenzi P., Leboffe L., Fiocchetti M., Nuzzo M.T., Brunori M (2016). Neuroglobin: from structure to function in health and disease. Mol. Aspects Med..

[3] Guimarães B.G., Hamdane D., Lechauve C., Marden M.C., Golinelli-Pimpaneau B (2014). The crystal structure of wild-type human brain neuroglobin reveals flexibility of the disulfide bond that regulates oxygen affinity. Acta Cryst. D Biol. Cryst..

[4] Pesce A., Dewilde S., Nardini M., Moens L., Ascenzi P., Hankeln T. (2003). Human brain neuroglobin structure reveals a distinct mode of controlling oxygen affinity. Structure.

[5] Ascenzi P., Brunori M (2016). A molecule for all seasons: the heme. J. Porphyr. Phthalocyanines.

[6] Holm L., Sander C (1993). Structural alignment of globins, phycocyanins and colicin A. FEBS Lett..

[7] Trent J.T., Watts R.A., Hargrove M.S (2001). Human neuroglobin, a hexacoordinate hemoglobin that reversibly binds oxygen. J. Biol. Chem..

[8] Dewilde S., Kiger L., Burmester T., Hankeln T., Baudin-Creuza V., Aerts T. (2001). Biochemical characterization and ligand binding properties of neuroglobin, a novel member of the globin family. J. Biol. Chem..

[9] Schmidt M., Giessl A., Laufs T., Hankeln T., Wolfrum U., Burmester T (2003). How does the eye breathe? Evidence for neuroglobin-mediated oxygen supply in the mammalian retina. J. Biol. Chem..

[10] Brunori M., Vallone B (2007). Neuroglobin, seven years after. Cell. Mol. Life Sci..

[11] De Simone G., Sbardella D., Oddone F., Pesce A., Coletta M., Ascenzi P (2021). Structural and (pseudo-)enzymatic properties of neuroglobin: its possible role in neuroprotection. Cells.

[12] Exertier C., Montemiglio L.C., Freda I., Gugole E., Parisi G., Savino C. (2022). Neuroglobin, clues to function and mechanism. Mol. Aspects Med..

[13] Pesce A., Nardini M., Bolognesi M., Bocedi A., Ascenzi P (2004). Structure‐function relationships in the growing hexa‐coordinate hemoglobin sub‐family. IUBMB Life.

[14] Kakar S., Hoffman F.G., Storz J.F., Fabian M., Hargrove M.S (2010). Structure and reactivity of hexacoordinate hemoglobins. Biophys. Chem..

[15] Nadra A.D., Martí M.A., Pesce A., Bolognesi M., Estrin D.A (2008). Exploring the molecular basis of heme coordination in human neuroglobin. Proteins.

[16] Brunori M., Vallone B (2006). A globin for the brain. FASEB J..

[17] Liu J., Yu Z., Guo S., Lee S.-R., Xing C., Zhang C. (2009). Effects of neuroglobin overexpression on mitochondrial function and oxidative stress following hypoxia/reoxygenation in cultured neurons. J. Neurosci. Res..

[18] Sun Y., Jin K., Peel A., Mao X.O., Xie L., Greenberg D.A (2003). Neuroglobin protects the brain from experimental stroke in vivo. Proc. Natl. Acad. Sci. U.S.A..

[19] Yu Z., Xu J., Liu N., Wang Y., Li X., Pallast S. (2012). Mitochondrial distribution of neuroglobin and its response to oxygen-glucose deprivation in primary-cultured mouse cortical neurons. Neuroscience.

[20] Antao S.T., Duong T.T., Aran R., Witting P.K (2010). Neuroglobin overexpression in cultured human neuronal cells protects against hydrogen peroxide insult via activating phosphoinositide-3 kinase and opening the mitochondrial K(ATP) channel. Antioxid. Redox Signal..

[21] Di Rocco G., Bernini F., Battistuzzi G., Ranieri A., Bortolotti C.A., Borsari M. (2023). Hydrogen peroxide induces heme degradation and protein aggregation in human neuroglobin: roles of the disulfide bridge and hydrogen-bonding in the distal heme cavity. FEBS J..

[22] Khan A.A., Mao X.O., Banwait S., Jin K., Greenberg D.A (2007). Neuroglobin attenuates beta-amyloid neurotoxicity in vitro and transgenic Alzheimer phenotype in vivo. Proc. Natl. Acad. Sci. U.S.A..

[23] Sun F., Mao X., Xie L., Greenberg David.A., Jin K (2013). Neuroglobin protein is upregulated in Alzheimer’s disease. J. Alzheimers Dis..

[24] Chen L.-M., Xiong Y.S., Kong F.L., Qu M., Wang Q., Chen X.Q. (2012). Neuroglobin attenuates Alzheimer-like tau hyperphosphorylation by activating Akt signaling. J. Neurochem..

[25] Li Y., Dai Y.B., Sun J.Y., Xiang Y., Yang J., Dai S.Y. (2016). Neuroglobin attenuates beta amyloid-induced apoptosis through inhibiting caspases activity by activating PI3K/Akt signaling pathway. J. Mol. Neurosci..

[26] Lee S., Van Bergen N.J., Kong G.Y., Chrysostomou V., Waugh H.S., O’Neill E.C. (2011). Mitochondrial dysfunction in glaucoma and emerging bioenergetic therapies. Exp. Eye Res..

[27] García-García F., Acosta-Hernández M.E., Beltrán-Parrazal L., Rodríguez-Alba J.C (2023). The role of neuroglobin in the sleep-wake cycle. Sleep Sci..

[28] Li W., Wu Y., Ren C., Lu Y., Gao Y., Zheng X. (2011). The activity of recombinant human neuroglobin as an antioxidant and free radical scavenger. Proteins.

[29] Herold S., Fago A., Weber R.E., Dewilde S., Moens L (2004). Reactivity studies of the Fe(III) and Fe(II)NO forms of human neuroglobin reveal a potential role against oxidative stress. J. Biol. Chem..

[30] Petersen M.G., Dewilde S., Fago A (2008). Reactions of ferrous neuroglobin and cytoglobin with nitrite under anaerobic conditions. J. Inorg. Biochem..

[31] Jin K., Mao X.O., Xie L., Khan A.A., Greenberg D.A (2008). Neuroglobin protects against nitric oxide toxicity. Neurosci. Lett..

[32] Brunori M., Giuffrè A., Nienhaus K., Nienhaus G.U., Scandurra F.M., Vallone B (2005). Neuroglobin, nitric oxide, and oxygen: functional pathways and conformational changes. Proc. Natl. Acad. Sci. U.S.A..

[33] Trashin S., de Jong M., Luyckx E., Dewilde S., De Wael K (2016). Electrochemical evidence for neuroglobin activity on NO at physiological concentrations. J. Biol. Chem..

[34] Tiso M., Tejero J., Basu S., Azarov I., Wang X., Simplaceanu V. (2011). Human neuroglobin functions as a redox-regulated nitrite reductase. J. Biol. Chem..

[35] Nicolis S., Monzani E., Ciaccio C., Ascenzi P., Moens L., Casella L (2007). Reactivity and endogenous modification by nitrite and hydrogen peroxide: does human neuroglobin act only as a scavenger?. Biochem. J..

[36] Brittain T., Skommer J., Raychaudhuri S., Birch N (2010). An antiapoptotic neuroprotective role for neuroglobin. Int. J. Mol. Sci..

[37] Brittain T (2012). The anti-apoptotic role of neuroglobin. Cells.

[38] Fago A., Mathews A.J., Moens L., Dewilde S., Brittain T (2006). The reaction of neuroglobin with potential redox protein partners cytochrome b5 and cytochrome C. FEBS Lett..

[39] Brittain T., Skommer J., Henty K., Birch N., Raychaudhuri S (2010). A role for human neuroglobin in apoptosis. IUBMB Life.

[40] Burmester T., Hankeln T (2014). Function and evolution of vertebrate globins. Acta Physiol. (Oxf)..

[41] Couture M., Burmester T., Hankeln T., Rousseau D.L (2001). The heme environment of mouse neuroglobin. Evidence for the presence of two conformations of the heme pocket. J. Biol. Chem..

[42] Hamdane D., Kiger L., Dewilde S., Green B.N., Pesce A., Uzan J. (2003). The redox state of the cell regulates the ligand binding affinity of human neuroglobin and cytoglobin. J. Biol. Chem..

[43] Ishikawa H., Kwak K., Chung J.K., Kim S., Fayer M.D (2008). Direct observation of fast protein conformational switching. Proc. Natl. Acad. Sci. U.S.A..

[44] Bellei M., Bortolotti C.A., Di Rocco G., Borsari M., Lancellotti L., Ranieri A (2018). The influence of the Cys46/Cys55 disulfide bond on the redox and spectroscopic properties of human neuroglobin. J. Inorg. Biochem..

[45] Vinck E., Van Doorslaer S., Dewilde S., Moens L (2004). Structural change of the heme pocket due to disulfide bridge formation is significantly larger for neuroglobin than for cytoglobin. J. Am. Chem. Soc..

[46] Fago A., Hundahl C., Malte H., Weber R.E (2004). Functional properties of neuroglobin and cytoglobin. Insights into the ancestral physiological roles of globins. IUBMB Life.

[47] Morozov A.N., Roach J.P., Kotzer M., Chatfield D.C (2014). A possible mechanism for redox control of human neuroglobin activity. J. Chem. Inf. Model..

[48] Lardinois O.M., Tomer K.B., Mason R.P., Deterding L.J (2008). Identification of protein radicals formed in the human neuroglobin-H2O2 reaction using immuno-spin trapping and mass spectrometry. Biochemistry.

[49] Schaffner I., Mlynek G., Flego N., Pühringer D., Libiseller-Egger J., Coates L. (2017). Molecular mechanism of enzymatic chlorite detoxification: insights from structural and kinetic studies. ACS Catal..

[50] Pfanzagl V., Bellei M., Hofbauer S., Laurent C.V.F.P., Furtmüller P.G., Oostenbrink C (2019). Redox thermodynamics of B-class dye-decolorizing peroxidases. J. Inorg. Biochem..

[51] Di Rocco G., Battistuzzi G., Borsari M., Bortolotti C.A., Ranieri A., Sola M (2021). The enthalpic and entropic terms of the reduction potential of metalloproteins: determinants and interplay. Coord. Chem. Rev..

[52] Milazzo L., Gabler T., Pühringer D., Jandova Z., Maresch D., Michlits H. (2019). Redox cofactor rotates during its stepwise decarboxylation: molecular mechanism of conversion of coproheme to heme b. ACS Catal..

[53] Gatin A., Duchambon P., Rest G.V., Billault I., Sicard-Roselli C (2022). Protein dimerization via tyr residues: Highlight of a slow process with co-existence of numerous intermediates and final products. Int. J. Mol. Sci..

[54] Bartesaghi S., Wenzel J., Trujillo M., López M., Joseph J., Kalyanaraman B. (2010). Lipid peroxyl radicals mediate tyrosine dimerization and nitration in membranes. Chem. Res. Toxicol..

[55] Nys K., Furtmüller P.G., Obinger C., Van Doorslaer S., Pfanzagl V (2021). On the track of long-range electron transfer in b-type dye-decolorizing peroxidases: identification of a tyrosyl radical by computational prediction and electron paramagnetic resonance spectroscopy. Biochemistry.

[56] Schmidt D., Falb N., Serra I., Bellei M., Pfanzagl V., Hofbauer S. (2023). Compound i formation and reactivity in dimeric chlorite dismutase: impact of pH and the dynamics of the catalytic arginine. Biochemistry.

[57] Chen Y.R., Chen C.L., Chen W., Zweier J.L., Augusto O., Radi R. (2004). Formation of protein tyrosine ortho-semiquinone radical and nitrotyrosine from cytochrome c-derived tyrosyl radical. J. Biol. Chem..

[58] Borsarelli C.D., Falomir-Lockhart L.J., Ostatná V., Fauerbach J.A., Hsiao H.H., Urlaub H. (2012). Biophysical properties and cellular toxicity of covalent crosslinked oligomers of α-synuclein formed by photoinduced side-chain tyrosyl radicals. Free Radic. Biol. Med..

[59] Chen P.Y., Funk M.A., Brignole E.J., Drennan C.L (2018). Disruption of an oligomeric interface prevents allosteric inhibition of Escherichia coli class Ia ribonucleotide reductase. J. Biol. Chem..

