# Supplementary material for: Insights into heme degradation and hydrogen peroxide-induced dimerization of human neuroglobin
Source: Biosci Rep. 2025 Jan 21;45(1):BSR20241265. doi: 10.1042/BSR20241265 (PMC12096953; doi:10.1042/BSR20241265)
Supplement: Figure S1 [file bsr-45-01-bsr-2024-1265-s001.docx]

**Supporting Information**

**Insights into heme degradation and hydrogen peroxide induced dimerization of human neuroglobin.**

**Alice Cassiani^1,2^, Paul G. Furtmüller^1^**, **Marco Borsari^2^,** **Gianantonio Battistuzzi^2,^*, Stefan Hofbauer^1,^***

^1^ *BOKU University, Department of Chemistry, Institute of Biochemistry, Muthgasse 18, A-1190, Vienna, Austria*

^2^ *Department of Chemical and Geological Sciences, University of Modena and Reggio Emilia, via Campi 103, 41125 Modena, Italy*

*****corresponding authors: Gianantonio Battistuzzi

Department of Chemical and Geological Sciences,

University of Modena and Reggio Emilia

via Campi n 103, 41126 Modena, Italy

tel: 059-2058639

e-mail: gianantonio.battistuzzi@unimore.it

ORCID: 0000-0003-4716-5745

Stefan Hofbauer

Department of Chemistry, Institute of Biochemistry

BOKU University

Muthgasse 18, 1190 Vienna, Austria

tel: +43 1 47654 77258

e-mail: stefan.hofbauer@boku.ac.at

ORCID: 0000-0003-3375-7715

**Keywords:** human neuroglobin, protein aggregation, heme bleaching, covalent link, oligomerization


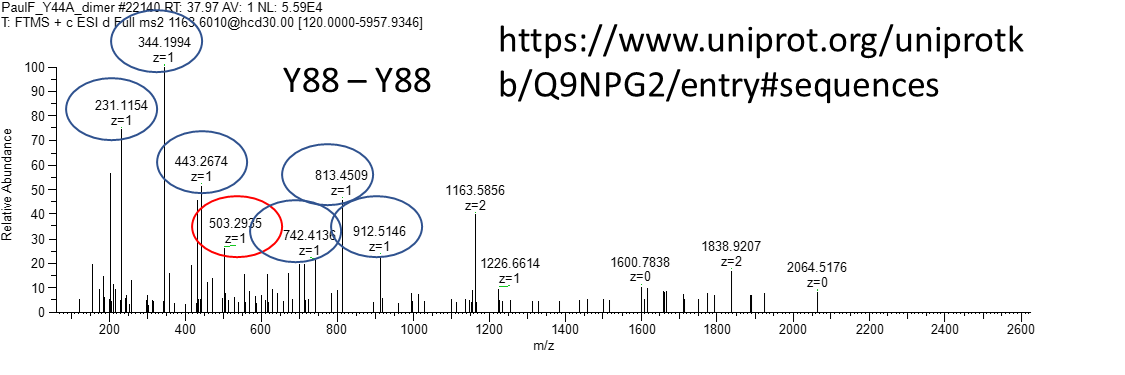


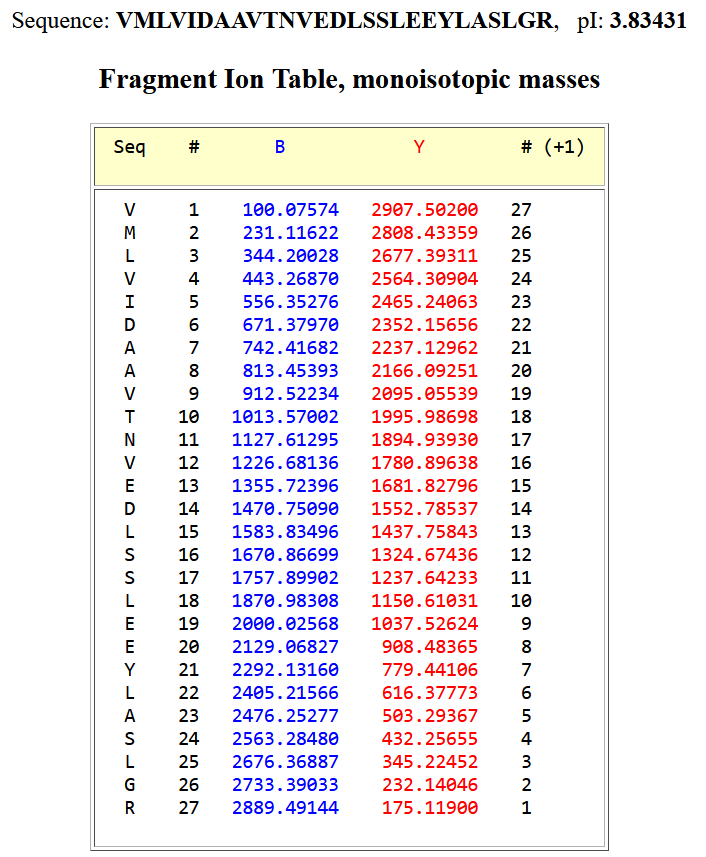


**Figure S1.** Representative MS2 spectrum of hNgb Y44A of the peptide with cross-linked Tyr88 residues. B-fragments are shown in blue, Y-fragments in red.
